# Effect of Chickpea (*Cicer arietinum* L.) Flour Incorporation on Quality, Antioxidant Properties, and Bioactive Compounds of Shortbread Cookies

**DOI:** 10.3390/foods13152356

**Published:** 2024-07-26

**Authors:** Katarzyna Felisiak, Sylwia Przybylska, Grzegorz Tokarczyk, Małgorzata Tabaszewska, Jacek Słupski, Joanna Wydurska

**Affiliations:** 1 Department of Fish, Plant and Gastronomy Technology, Faculty of Food Sciences and Fisheries, West Pomeranian University of Technology, 70-310 Szczecin, Poland; sylwia.przybylska@zut.edu.pl (S.P.); grzegorz.tokarczyk@zut.edu.pl (G.T.);; 2 Department of Plant Product Technology and Nutrition Hygiene, Faculty of Food Technology, University of Agriculture in Kraków, 30-149 Kraków, Poland; malgorzata.tabaszewska@urk.edu.pl (M.T.); jacek.slupski@urk.edu.pl (J.S.)

**Keywords:** chickpea, flour replacement, antioxidant activity, polyphenols

## Abstract

High nutritional value and antioxidant properties make chickpea flour a valuable substitute for wheat flour, although its texture-forming abilities are different. The aim of this study was to investigate the possibility of increasing the content of bioactive compounds and antioxidant properties of shortbread cookies by simple partial or complete replacement of wheat flour with chickpea flour without considerable changes in texture, color, sensory properties, or acceptability. Shortbread cookies were made from wheat flour (0% of chickpea flour), wheat flour and chickpea flour (replacement of 25%, 50%, and 75%), and chickpea flour (100%). Generally, the increase in chickpea flour share resulted in an increase in protein, fat, and ash content, as well as antioxidant properties. Polyphenol content, flavonoid content, and antioxidant activities increased three- to sixfold in shortbread cookies containing chickpea flour in comparison to wheat cookies. The level of proteins increased about 50% and the antioxidant properties were three to six times higher than in wheat cookies. Cookies containing up to 75% chickpea flour were assessed as very good or good quality, while only cookies without wheat flour were assessed as sufficient quality. It could be concluded that part of the wheat flour content in shortbread cookies can be replaced by chickpea flour. Application of a 25% proportion of chickpea flour increases physicochemical properties without changes in sensory properties. Sensory quality was up to 75% lower, but antioxidant properties were increased. However, complete replacement of wheat flour in shortbread cookies without changing the recipe resulted in a product of slightly lower sensory quality.

## 1. Introduction

Chickpea (*Cicer arietinum* L.) is a widely consumed legume in the world, especially on the India and Mediterranean Sea coasts [1]. It requires high temperature in cultivation, but does not require high humidity; therefore, it is a plant suitable for problems with rainfall [2,3]. This is a cheap source of proteins compared with animal sources, which is important for low-income people in many countries [2]. Chickpea proteins, 20–22 g/100 g d.m., are rich in essential amino acids, including lysine (1.2–1.4 g/100 g d.m.), which is a limiting amino acid for wheat products. In contrast, the limiting amino acids in chickpea proteins are methionine and cysteine, sulfur-containing amino acids that are present at the level of 0.22–0.27 g/100 g d.m. [4]. Moreover, chickpea is a rich source of carbohydrates (60–65%), including non-digestible oligosaccharides and resistant starch. It also contains high amounts of minerals, especially alkaline, unsaturated fatty acids, carotenoids, and tocopherols, and polyphenols, including flavonoids, proanthocyanidins, hydroxybenzoic acids, and hydroxycinnamic acids, as well as isoflavones [1,5,6,7,8,9]. These compounds are important for health because of their antioxidant properties, anti-inflammatory, anticancerogenic, and antimutagenic properties, among others [9,10]. Chickpeas also contain some antinutritional compounds, which may have a negative effect on the body, including trypsin inhibitors, α-amylase inhibitors, raffinose, saponins, tannins, and phytates [9,11]. Some of these components do not pose a danger in thermally processed products, which are inactivated by heat treatment. In turn, reduction in phytates and tannins can be achieved by dehulling, soaking, and roasting [11,12]. However, it has been found that non-digestible oligosaccharides, which are usually classified as antinutritional ingredients, are fermented by microorganisms in the digestive system, so they act as prebiotics [11]. Tannins, which form complexes with proteins, have a simultaneous antioxidant effect [9].

Chickpeas are used in many dishes, and chickpea flour is also popular. It has a lower glycemic index than wheat flour, which is important to diabetics and people with insulin resistance [13,14]. People consuming a diet rich in chickpeas (as cooked seeds and bread or biscuits made from wheat and chickpea flour blend) have significantly lower fluctuations in blood glucose levels and lower insulin levels [15,16,17,18]. Therefore, increasing the intake of chickpea products may be beneficial.

In the last few years, research on the possibility of wheat flour replacement in bakery products was carried out. Corn flour or rice flour are the main substitutes for wheat flour used in the preparation of biscuits, cakes, and pasta, among others [19,20,21]. Other gluten-free flours have been tested by researchers, for example, plantain flour [22], quinoa flour [23], Amaranthus flour, pumpkin seed flour [24,25], and buckwheat flour [26], as well as flour blends [27]. Thongram et al. [28] studied partial replacement of wheat flour in biscuits by pulse flours (25%), namely, chickpea flour, pigeon pea, mung bean flour, cowpea flour, and flour blends containing flours from four pulses (10% each). Pasta with 5–10% addition of chickpea flour additions still met the specification of pasta products, but 15% decreased elasticity and sensory properties of cooked products [14,29,30]. In gluten-free bread, chickpea flour increased nutritional value, but had lower bread specific volume [31,32]. In turn, instant noodles with chickpea flour addition up to 25% had higher levels of essential amino acids and good sensory properties [33]. Gluten-free crackers made from chickpea flour scored well during consumer acceptance testing [34]. On the other hand, the texture of chickpea muffins was improper, but flour blends containing chickpea flour and corn starch (50:50) or chestnut flour (75:25) resulted in cookies with much better sensory properties [35,36].

Cakes and cookies are bakery and confectionery products that account for a large share of the food market. Cookie sales have been increasing in recent years, especially in North America, Europe, and Asia, and the global market shows an annual growth rate of 5.3% [37]. One of the most popular types of cookies are shortbread cookies. They are dry, bristle, and durable products that contain a lot of fat. Thanks to the presence of solid fat, the gluten in the flour does not develop; therefore, these cookies are crumbly and fall apart when bitten. Consumers expect that their preferred products will have high sensory quality and at the same time health-promoting properties [38]. Nevertheless, replacing wheat flour with another kind of flour may bring problems with product texture and cause hardness or excessive looseness in shortbread cookies [28]. Although there has been some research on chickpea flour application in different cookies, mixtures of chickpea flour and, for example, corn flour or chestnut flour [35,36] were used and required different technologies [39]. Furthermore, there were either The nutritional value, texture parameters, and sensory properties of ready-made products and bioactive compounds and antioxidant properties of chickpea seeds or chickpea flour [11] have been determined. To the best of the authors’ knowledge, there are no studies to date on replacing wheat flour with chickpea flour in shortbread cookies to take into account all these aspects at the same time.

Therefore, the aim of the study was to investigate the possibility of increasing the content of bioactive compounds and antioxidant properties of shortbread cookies by simple partial or complete replacement of wheat flour with chickpea flour while maintaining the desired texture, color, and sensory quality.

## 2. Materials and Methods

### 2.1. Materials and Reagents

All materials used to prepare shortbread cookies were purchased from a local market (Szczecin, Poland) in February 2023. Wheat flour type 450 (fine white flour, granulation 90–130 µm, GoodMills Poland Sp. z o.o., Stradunia, Poland), chickpea flour (granulation 180–300 µm; chickpea seeds of Kabuli type planted in Turkey, dehulled and milled by Ebro Ingredients, Wormer, Netherlands; confected by Melvit, Kruki, Poland), whole hen eggs (approx. 50 g each; A class, size M, Czachorowski, Gdańsk, Poland), icing sugar (white, granulation 80–100 µm, Diamant, Pfeifer & Langen Marketing Sp. z o.o., Poznań, Poland), table salt (fine, grains 0.5–2 mm, refined, Kłodawa, Poland) and margarine (80% fat, hydrogenated vegetable oils: palm, rapeseed and sunflower, Bielmar, Bielsko Biała, Poland) were used in the study.

Reagents: 2,2′-azinobis-(3-ethylbenzothiazoline-6-sulfonic acid (ABTS), 6-hydroxy-2,5,7,8-tetramethylchroman-2-carboxylic acid (Trolox), 2,2-diphenyl-1-picrylhydrazyl (DPPH), 2,4,6-Tris(2-pyridyl)-s-triazine (TPTZ), 3-(2-pyridyl)-5,6-diphenyl-1,2,4-triazine-4′,4′′-disulfonic acid sodium salt (ferrozine), gallic acid (GA), ethylenediaminetetraacetic acid (EDTA), and aluminum chloride anhydrous were obtained from Sigma-Aldrich (Germany). Iron (II) chloride tetrahydrate (FeCl_2_·4H_2_O) was obtained from VWR Chemicals (Belgium). Iron (III) chloride hexahydrate FeCl_3_·6H_2_O), potassium persulfate (K_2_S_2_O_8_), Folin–Ciocâlteu reagent, sodium carbonate anhydrous, pure methanol, hydrochloric acid, anhydrous sodium acetate, pure glacial acetic acid, sodium hydroxide, and sulfuric acid (VI) were obtained from Chempur (Piekary Śląskie, Poland) and 5-sulphosalicylic acid dihydrate was obtained from POCh (Radom, Poland). All chemicals were of analytical grade.

### 2.2. Preparation of Shortbread Cookies

Shortbread cookies were produced according to the following recipe, based on a modified traditional recipe for short-crust pastry [40] (Table 1). Cold (5 ± 1 °C) margarine was cut into cubes and mixed quickly with other ingredients in a planetary mixer (KitchenAid, Benton Harbor, Michigan, USA). Shortbread dough was formed into a cylinder, wrapped in cling film, and placed in the refrigerator for 1 h. Then, the pastry was rolled out to a thickness of 4 mm, circles were cut out using a 5 cm-diameter cutter, and each was weighed. They were placed on a baking tray lined with baking paper and baked at a temperature of 170 ± 2 °C in a convection oven (Hendi H90S, Rhenen, Germany) for 14 min. Afterwards, cookies were transferred to a cake rack and cooled, then they were weighed and measured. Cold cookies were packed into plastic boxes, covered, and stored at ambient temperature (20 ± 1 °C) for 24 h until testing.

### 2.3. Sensory Evaluation

The sensory evaluation of cookies was carried out with the participation of a 10-person team of experts (5 men and 5 women) aged 25 to 50. Informed consent was obtained from all subjects involved in the study.

The sensory panel was trained in sensory sensitivity [41,42]. The assessment was performed by everyone at an individual station, under standard lighting conditions, temperature of 20 ± 1 °C, and humidity of 75% [43] in three independent repetitions. Unit samples of shortbread cookies were placed in previously prepared and coded plastic containers and closed with a lid. Boiled water at a temperature of 40 °C was used as a taste neutralizer between the evaluation of the analyzed baked goods. After opening the package, each coded sample was organoleptically assessed: appearance, color, taste, odor, and texture on a 5-point scale (1—bad, 2—unsatisfactory, 3—satisfactory, 4—good, 5—very good) [44].

For a more detailed characterization of the taste and odor characteristics of the obtained cookies, 9 taste characteristics (sweet, nutty, chickpea, egg, burnt, rancid, bitter, bland, foreign) and 8 odor characteristics (typical, nutty, chickpea, egg, burnt, rancid, butter, foreign) on a 6-point scale (0—imperceptible, 1—very weak, 2—weak, 3—moderate, 4—distinct, 5—very distinct) were used [44].

Additionally, the desirability of cookies was assessed on a 9-point hedonic scale in accordance with the ISO 4121:2003 standard [45] (1—extremely undesirable, 2—very undesirable, 3—undesirable, 4—somewhat undesirable, 5—neither undesirable nor desirable, 6—somewhat desirable, 7—desirable, 8—very desirable, 9—extremely desirable).

The results of the assessments were recorded on assessment cards prepared for each person (I—organoleptic assessment, II—assessment of taste and odor characteristics, III—desirability assessment). From card I, after the analysis and decoding of the samples, the overall rating of cookies was calculated as a weighted average of the individual partial ratings, taking into account the weighting factors: appearance—0.1, color—0.1, odor—0.2, texture—0.3, and taste—0.3. This allowed each of them to be assigned to one of the quality classes: 5–4.5 points—very good quality, 4.4–3.5 points—good quality, 3.4–2.6 points—satisfactory quality, 2.5–1.6 points—insufficient quality, <1.5 points—poor quality [46]. From card II, sensory profiles were determined based on the calculated average value for individual taste and odor characteristics, and from card III, the level of desirability of the tested shortbread cookies was determined.

### 2.4. Color Parameter Analysis

The color parameters analysis of shortbread cookies was carried out on an NH 310 colorimeter (Shenzhen Technology Co., Ltd., Guangdong, China) in a CIE Lab system. Register of color components (L*—lightness—on a scale from 0 (perfect black) to 100 (perfect white), a*—coordinate of red and green (a* > 0—redness; a* < 0—greenness) and b*—yellow and blue coordinate (b* > 0—yellowness; b* < 0—blueness)) was performed in seven replicates for each sample. In addition to the basic color parameters, saturation C* (color intensity) and tone angle h* (hue) were measured [47,48]. The overall color difference *ΔE* was determined between the control sample without the addition of chickpea flour and the sample with its addition. The color of shortbread cookies (outer part) was measured pointwise at room temperature (20 ± 1 °C), by direct touching with the measuring aperture at a diameter of 8 mm. The measurements of the samples were carried out with SCI geometry using a D65 illuminator and CIE 10° standard observer. Directly before the actual determination of the L*, a*, b*, C*, h* and *ΔE* color components of shortbread cookies, the apparatus was calibrated on a white and black standard. The mean value and standard deviation were calculated from the obtained values.

The total color difference (*ΔE*) was interpreted according to the following criteria: 0–0.5—no difference, 1–1.5—slight difference; 2–3—noticeable difference; 3.5–4—a clear difference and >5—a very clear difference [49,50]. In order to determine the degree of color change in the surface of the products on the basis of the measured values of L*, a* and b* parameters, the browning index (*BI*) was determined. The following equations were used to calculate this indicator [51]:

$$BI\;=\frac{\left[\left(X-0.31\right)\cdot 100\right]}{0.17}$$

where $X=\frac{a^{*}+1.75\cdot L^{*}}{5.645\cdot L^{*}+a^{*}-3.012\cdot b^{*}}$

### 2.5. Texture Parameter Analysis

The textural attributes of the samples were instrumentally measured within six individual cookies (six repetitions) with TA using an XT Plus C^®^ Texture Analyzer (Stable Micro Systems, Godalming, UK). All experiments were conducted in a controlled temperature room at 20 ± 1 °C. The texture analyzer settings were as follows: pretest speed: 1.5 mm/s; test speed: 1.0 mm/s; posttest speed: 1.5 mm/s; trigger force: 5 g; trigger type: auto; data acquisition rate: 200 points per second (pps) [52]. TPA analyses were performed 24 h after preparation of cookies, which were stored in closed plastic boxes.

The hardness and crispness were measured with a texture analyzer equipped with a 3-point bend rig (HDP/3PB) according to the method described by Krzywiński et al. [52]. Hardness was indicated by the maximal strength needed to break the product (top of peak), whereas crispness indicated by the first upper peak of the load during compression of the sample at the moment of breaking its structure [53,54].

### 2.6. Water Activity

Water activity (a_w_) was determined in triplicate using a HygroLab C1 instrument (Rotronic, Bassersdorf, Switzerland) equipped with an HC2-AW probe [55] calibrated in the range 0.1–0.95 with solutions of LiCl of known activity [56]. Each sample was measured by covering the bottom of a plastic disposable cup with a small portion of milled shortbread cookies, placing the cup into the sample holder, and taking the reading.

### 2.7. Water Absorption Capacity (WAC) and Oil Absorption Capacity (OAC) of Flours

Water absorption capacity (WAC) of flours was determined according to Chandra et al. [57]. Briefly, 10 mL of distilled water was added to 1 g of flour and mixed. After 30 min, samples were centrifuged (MPW-351, MPW Med. Instruments, Warsaw, Poland) at 2490× *g* for 20 min at 20 ± 1 °C, the supernatants were discarded, and after removing liquid, the residual was weighted. WAC was calculated as grams of water per gram of flour.

Oil absorption capacity (OAC) of flours was determined according to Chandra et al. [57]. Briefly, 10 mL of refined rapeseed oil was added to 1 g of flour and mixed. After 30 min, samples were centrifuged (MPW-351, MPW Med. Instruments, Warsaw, Poland) at 2490× *g* for 20 min at 20 ± 1 °C, the supernatants were discarded, and after removing liquid, the residual was weighted. WAC was calculated as grams of oil per gram of flour.

### 2.8. Extract Preparation

Methanolic extracts were obtained by shaking milled samples in 80% aqueous methanol (ratio 1:10, *w*/*v*) for 2 h in laboratory platform shaker WL-1 (JW Electronic, Warsaw, Poland) in a linear motion, speed 100 rpm, according to Michalska et al. [58]. After filtration through qualitative filter paper, the extracts were collected in dark bottles. Methanolic extracts were used for total polyphenol, total flavonoid content, and antioxidant activity determinations.

Ether extracts were prepared according to Rodriguez-Amaya and Kimura [59] with some modifications. After grinding cookies in a mortar, 2 g of each sample was transferred to a dark glass bottle. Acetone (10 mL) was added, and the bottle was closed and shaken for 15 min in an Ultron ultrasonic device (Dywity, Poland). The extraction was repeated twice with a fresh portion of acetone until the sample was completely discolored. The collected acetone extract was transferred to a separator and the sample was purified from acetone by adding petroleum ether (20 mL) and then distilled water to separate the two phases. After discarding the water–acetone phase, the obtained ether extract was passed through anhydrous sodium sulfate. Ether extracts were used for total carotenoid content determination.

Extracts for phytate determination were prepared by shaking milled samples with 2.4% HCl solution (ratio 1:50, *w*/*v*) for 1 h in a laboratory platform shaker WL-1 (JW Electronic, Warsaw, Poland) in a linear motion at 100 rpm and 20 ± 1 °C, as per Laelago et al. [12]. Then, samples were centrifuged at 2490× *g* for 15 min at 20 ± 1 °C (MPW-351, MPW Med. Instruments, Warsaw, Poland) and the supernatants were collected.

### 2.9. Chemical Determinations

All spectrophotometric determinations were conducted using a UV-vis Helios γ Spectrophotometer (Thermo Spectronic, Horsham, UK). Extracts and reagents were mixed using a vortex shaker (IKA, Warsaw, Poland). Centrifugation was conducted using an MPW-351 Centrifugator (MPW Med. Instruments, Warsaw, Poland). All analyses were performed in three replications.

#### 2.9.1. Composition

Total nitrogen, fat, moisture, and ash content were determined by AOAC methods to be 950.36, 922.06, 925.10, 923.03, respectively [60]. Carbohydrate content was calculated as the difference between 100 and the sum of other constituents. Starch plus sugar content was calculated as the difference between total carbohydrates and total dietary fiber content.

#### 2.9.2. Fiber Content

The content of soluble (SDF) and insoluble dietary fiber (IDF) was determined according to AOAC methods (992.16) [61] using enzyme kits and procedures from Megazyme (Megazyme International Ireland Ltd.—K-TDFR 12/05, Wicklow, Ireland). The ground samples were degreased using petroleum ether and then subjected to the action of thermostable α-amylase, protease, and amyloglucosidase. The samples were filtered through Schott crucibles to determine insoluble fiber. Soluble fiber was precipitated with 95% ethanol and filtered on a Schott crucible. The precipitate was dried at 103 °C to a constant weight and weighed. Then, the ash content of the dried sample was determined by ashing the sample in a Nabertherm GmbH L9/S (Lilienthal, Germany) oven at 525 °C for 5 h and the protein content was determined using the Kjeldahl method. The dietary fiber content was the product of the dilution and the weight of the sediment on the Schott crucible minus the protein and ash content.

#### 2.9.3. Bioactive Compound Content

Total phenolic compounds (TPCs) were determined in methanolic extracts with Folin–Ciocâlteu reagent [62]. Briefly, 5 mL of 10% Folin–Ciocâlteu reagent (*v*/*v*) was added to 1 mL of diluted samples, and after 5 min, 4 mL of 7.5% sodium carbonate solution (*w*/*v*) was added and vortexed. Test tubes were incubated at room temperature in the dark for 2 h. Then, absorbance was measured at 750 nm. Results are expressed as gallic acid equivalents per gram of cookies wet weight (mg GAE/g).

Total flavonoid content (TFC) was determined according to Shraim et al. [63]. Briefly, 2 mL of fourfold-diluted methanolic extract was mixed with 0.1 mL of 10% aluminum chloride (*w*/*v*), 0.1 mL 1 M sodium acetate, and distilled water to obtain 5 mL. After 30 min, the absorbance was measured at 415 nm. Results are expressed as quercetin equivalents per gram of cookies wet weight (mg QE/g).

Total carotenoid content (TC) in the analyzed cookies was determined in ether extracts prepared according to Rodriguez-Amaya and Kimura [59] with modifications as described above (point 2.8). The absorbance of the extracts, prepared in triplicate for each sample, was measured at a wavelength of 450 nm. The content of total carotenoids was calculated according to the formula of Alam et al. [64], and is expressed in micrograms/100 g.

#### 2.9.4. Antioxidant Activities

The antioxidant activity of cookies was determined according to Re et al. [65] as the Trolox equivalent antioxidant capacity (TEAC) of methanolic extracts. ABTS cation radical was prepared as water solution of 7 mmol/L ABTS activated with K_2_S_2_O_8_ (2.45 mmol/L) for 16 h. Directly before analysis, stock solution was diluted with methanol to absorbance of 0.70 ± 0.02 at 734 nm. Then, 40 µL of methanolic extract and 4 mL of ABTS^•+^ solution were mixed and incubated in the dark for 30 min. Afterward, the absorbance was measured at 734 nm. TEAC was calculated as decrease in the absorbance and compared to the action of Trolox, expressed as Trolox equivalents per 1 g of cookies wet weight (µmol TE/g).

The radical scavenging activity (RSA) was determined in methanolic extracts according to Brand-Williams et al. [66]. Diluted samples (4 g) were mixed with 1 mL of 0.2 mmol/L DPPH methanol solution and the absorbance was measured at 517 nm after 30 min incubation in the dark. RSA was calculated as the decrease in the absorbance (in the range of 20–80%) and related to the action of Trolox against the free radical DPPH (based on the standard curve), expressed as Trolox equivalents per gram of cookies wet weight (µmol TE/g).

Ferric reducing antioxidant power (FRAP) was determined by mixing 100 µL methanolic extracts with 3 mL of reagent (prepared freshly by combining TPTZ in 0.04 mol/L HCl, 0.02 mol/L FeCl_3_ solution and 0.3 mol/L acetic buffer pH 3.6 at 1:1:10, and heated at 37 °C for 30 min) according to Benzie and Strain [67]. Samples were mixed and incubated at room temperature for 30 min, then the absorbance was measured at 593 nm. Results are expressed as Trolox equivalents (µmol TE) per gram of wet weight of cookies.

Ferrous chelating ability (FCA) was determined in methanolic extracts according to Khantaphant et al. [68]. Briefly, 0.5 mL of extract and 3 mL of distilled water were mixed with 0.01 mL of 2 mM FeCl_2_ and 0.02 mL of 5 mM ferrozine. After 20 min in the dark, the absorbance was measured at 562 nm, and the results are expressed as EDTA equivalents (µmol EDTA) per gram of cookies wet weight.

#### 2.9.5. Phytate Content

For phytate determination, 3 mL of hydrochloride extract was mixed with 1 mL of Wade reagent (containing 0.03% FeCl_3_ solution and 0.3% sulfosalicylic solution in water), vortexed as above, and absorbance determined at 500 nm [12]. Results are expressed as phytic acid equivalents (mg PA/g).

### 2.10. Chromatographic Analyses

Chromatographic analyses were carried out using a Dionex UltiMate 3000 HPLC set with a DAD detector from Thermo Scientific (Germering, Germany). The column used was a Cosmosil 5C18–MS-II 250 × 4.6 mm ID, 5 μm from Nacalai Tesque, INC. (Kyoto, Japan).

#### 2.10.1. Determination of Phenolic Compounds by HPLC (Polyphenol Profile)

This determination was made based on the method described by Klimczak et al. [69] modified by Tabaszewska and Najgebauer-Lejko [70]. The crushed samples were collected into Eppendorf centrifuge tubes, then HPLC methanol with 1% L-ascorbic acid (*v*/*w*) was added to them. The samples were mixed using a vortex (Labnet, Edison, NJ, USA), then sonicated for 15 min, 20 °C, and then centrifuged for 20 min, 30,065× *g* at 4 °C (MPW—260R centrifuge, Warsaw, Poland). The supernatant was filtered using PTFE-L syringe filters with a pore diameter of 0.22 μm. Analyses lasted 50 min at a flow rate of 1 mL/min. The mobile phase included: A—2% aqueous acetic acid solution and B—100% methanol. The eluent system was as follows: eluent A—10 min 70%; 25 min 50%; 35 min 30%; 40 min 95%; and 95% until the end of the analysis, at wavelengths λ = 245 nm, λ = 280 nm, λ = 320 nm, and λ = 360 nm. The determination was performed in four repetitions. The compounds were identified by retention time and spectra, and the content was calculated based on standard curves. Calibration curves were prepared for the following standards: caffeic acid, vanillic acid, protocatechuic acid, ferulic acid, rutin, kaempferol, (+) catechin, (−) epicatechin, syringic acid (Sigma Aldrich, Guangzhou, China), salicylic acid (Chempur, Piekary Śląskie, Poland), *p*-coumaric acid, ellagic acid (Sigma Aldrich, Gillingham, UK), quercetin, synaptic acid (Sigma Aldrich, Bangalore, India), chlorogenic acid (Sigma Aldrich, Buchs, Switzerland), hippuric acid, apigenin (Sigma Aldrich, Taufkirchen, Germany), (±) naringenin (Sigma Aldrich, Gillingham, UK), phlorizin, 3-hydroxy-benzoic acid (Sigma Aldrich, St. Louis, MO, USA), malvidin chloride, myricetin (Sigma Aldrich, St. Quentin Fallavier, France), gallic acid, 4-hydroxy-benzoic acid (Merck, Darmstadt, Germany), *t*-cinnamic acid (Loba, Fischamend, Austria), all of HPLC grade.

#### 2.10.2. Determination of Hydroxymethylfurfural (HMF) Content

Samples for analysis were prepared based on Tomf-Sarna [71] with modification. The crushed samples were collected in Eppendorf centrifuge tubes and then deionized water was added to them. The samples were sonicated for 5 min, temperature 20 °C, and then centrifuged for 15 min, 18,228× *g*, temperature 4 °C (MPW—260R centrifuge, Warsaw, Poland). The supernatant was filtered using Teflon syringe filters with a pore diameter of 0.45 μm. Chromatographic analysis was performed based on the method described in PN-EN 14177 [72] in isocratic flow of two eluents: A—water (90%) and B—acetonitrile (10%) at a flow rate of 1 mL/min. Analysis lasted 15 min, and wavelength was 276 nm. The assay was performed in four repetitions. HMF was identified by retention time and the content was calculated based on a standard curve (regression equation y = 2.4518x).

### 2.11. Statistical Analysis

Analyses were performed in three replications, unless otherwise specified. Results were analyzed statistically using one-way analysis of variance (ANOVA) with StatSoft Statistica 13.3 software (Statsoft, Tulsa, OK, USA). An ANOVA *p* value was set at 0.05, and significant differences between samples were examined using the post hoc Tukey’s test (*p* < 0.05).

Principal component analysis (PCA) and cluster analysis were performed to ascertain correlations between components of cookies, bioactive compounds, and antioxidant activities, browning index and texture parameters, and overall sensory assessment of shortbread cookies containing chickpea flour.

## 3. Results and Discussion

### 3.1. Sensory Properties of Shortbread Cookies

**Figure 1:**
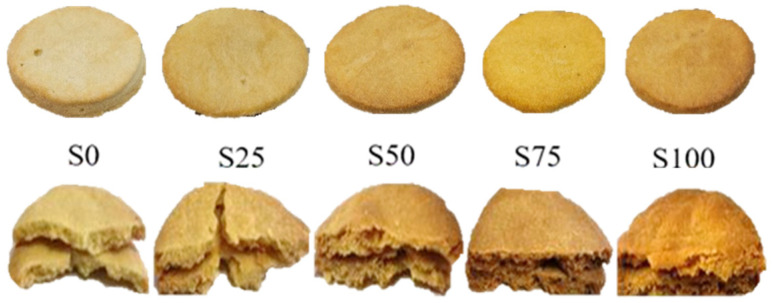
Effect of chickpea flour on the appearance and color of shortbread cookies (share of chickpea flour: S0—0%, S25—25%, S50—50%, S75—75%, S100—100%). Photo J. Wydurska.

Wheat flour and chickpea flour were used to obtain shortbread cookies. Chickpea flour was much darker than wheat flour (Figure S1) and differed in composition and properties. The use of chickpea flour caused darker and uneven surfaces of the cookies (Figure 1).

During sensory assessment, the shortbread cookies containing chickpea flour were found to be crunchier and drier than wheat cookies, but they were still rated very well and well by the evaluation panel (Table 2).

**Figure 2:**
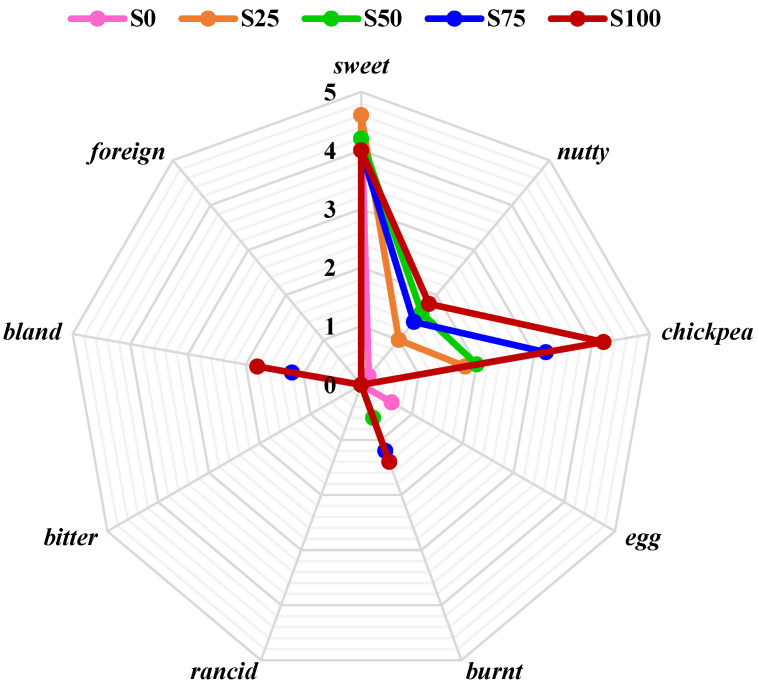
Taste sensory profile of shortbread cookies without and with chickpea flour.

**Figure 3:**
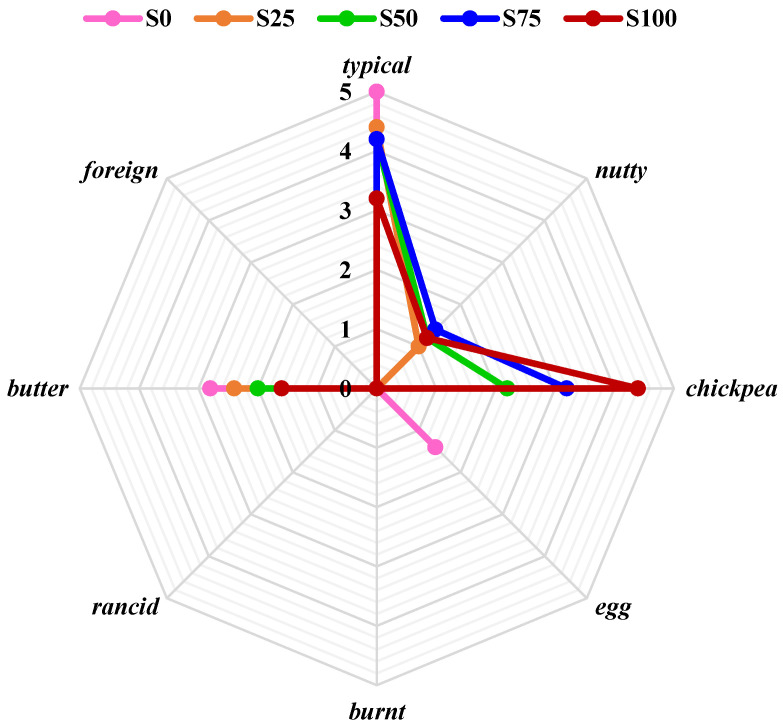
Odor sensory profile of shortbread cookies without and with chickpea flour.

Addition of chickpea flour influenced the odor and taste slightly, as well as the texture. However, the desirability of cookies containing both flours was very high (7.0 to 7.8 points). The slightly negative influence on taste, aroma, and texture was noticed only in sample S100. Although the notes of cookie taste differed statistically significantly (*p* < 0.05) with increasing chickpea flour above 25% (S50 and S75), cookies were characterized as good quality. In cookies containing 100% chickpea flour, overall desirability was a little bit lower (5.8) and the quality described as sufficient (Table 1). Their taste and odor were less typical: a hint of chickpea and nutty taste and odor were clearly felt (Figure 2 and Figure 3). Thongram et al. [28] also found that the addition of 25% chickpea flour instead of wheat flour did not cause significant differences (*p* > 0.05) in organoleptic quality. Similar effects were reported by Yadav et al. [22], who studied the influence of a plantain and chickpea flour blend addition on biscuit quality. They found that replacing wheat flour with up to 20% of plantain flour and 20% of chickpea flour (wheat flour share—60%) did not influence significantly the sensory properties of the biscuits, but a higher share of flour blend caused decreases in all sensory parameters and overall acceptability. Dogruer et al. [73] compared several types of chickpea flours and found medium quality (5 points on a 7-point scale), especially cookies made from germinated chickpea flour.

Increase in chickpea flour share resulted in sweet and egg tastes being less intensive. Nutty, chickpea, bland, and burnt taste were perceptible, although not intensive (e.g., burnt 1.4 from 5). At the same time, the perceptibility of egg odor and butter odor decreased, and nutty and chickpea odor increased (Figure 2 and Figure 3).

### 3.2. Color Parameters Analysis

The flours used in the production of shortbread cookies—wheat and chickpea flour—differed significantly (*p* < 0.05) in the assessed color parameters (Table S1). Chickpea flour was creamy in color and wheat flour was white, which is confirmed by the designated color indices (L*, a* and b*). The lightness (L*) of wheat flour was 4% higher, while its redness (a*) and yellowness (b*) were 57% and 47% lower, respectively, than parameters of chickpea flour.

The lightness parameter (L*) of shortbread cookies decreased from 67.6 (S25) to 49.7 (S100) with increasing share of chickpea flour (Table 3). For S0, parameter L* was 58.9 and did not differ (*p* > 0.05) from samples S50 and S75. Similarly, a study by Lu et al. [39] showed that the addition of chickpea flour (from 10% to 40%) caused a decrease in the color lightness in biscuits compared to a control sample (without its addition) and an increase in the a* parameter (redness). In our research, the redness of cookies ranged from 10.6 (S25) to 16.9 (S75). Sample S0, whose a* parameter reached a value of 11.1, differed (*p* < 0.05) from samples S50–S100. The addition of chickpea flour also increased the yellowness (b*) of cookies from 23.0 (S25) to 31.1 (S100). In S0, parameter b* reached a value of 22.2 and differed (*p* < 0.05) from cookies in which chickpea flour constituted 50%, 75% and 100%. In a study by Torra et al. [36], the addition of chestnut flour to chickpea flour in cookies also reduced the L* parameter and increased the a* parameter, but decreased the b* parameter, which may be due to the composition of the component used. On the other hand, studies by Demirkesen [74] showed a similar course of changes in the color parameters L*, a* and b* in cookies made from chickpea flour with the addition of rice flour. In the assessment of color saturation C*, all cookies differed (*p* < 0.05) except S25 (*p* > 0.05). The parameter h* (tone angle), on the contrary, did not differ in any samples except for S25 (*p* < 0.05). The overall color difference *ΔE* in S25 was very visible (*ΔE* = 8.01). Among all cookies, the greatest *ΔE* parameter was noted in S75 (14.42). For comparison, S100 was characterized by 15% lower *ΔE* than S75.

The darkening index (BI) determined for tested cookies ranged from 48.65 (S25) to 105.12 (S75) (Table 3). The addition of 50%, 75%, and 100% of chickpea flour had a similar effect on the values of BI without any noticeable differences between samples (*p* > 0.05), and they differed from cookies S0 and S25 (*p* < 0.05). In S0, the browning index was 58.73 and differed from all other cookies (*p* < 0.05).

According to Chevalier et al. [75], the color of cookies depends mainly on the Maillard reactions that occur between proteins and reducing sugars and on the caramelization of sugar. As confirmed by Torra et al. [36] and Schouten et al. [76], chickpea flour, due to the high content of proteins and amino acids, has a significant impact on the formation of Maillard reaction products during baking. Similarly, research by Singh et al. [77], showed that simultaneously with the increase in protein content in muffins with the addition of dark bean flour, the color became darker as a result of the Maillard reaction. The intensity of the reaction and the increase in the BI index in cookies resulted from the specificity of the product and factors flavoring them [78]. Our research confirms that addition of chickpea flour caused and increase in BI; however, cookies with 50–100% share did not differ statistically significantly (*p* < 0.05).

The overall color differences (*ΔE*) of cookies were influenced by share of chickpea flour (*p* < 0.05). The *ΔE* value was 8.01 for S25, which, according to the criterion provided by Adekunte et al. [49] and Mokrzycki and Tatol [50], is interpreted as a very clear difference. The increased share of chickpea flour from 50% to 100% caused an increase in the *ΔE* parameter from 10.1 to 14.42 and more visible differences in color. In the case of S100, the *ΔE* reached a lower value than S75 (12.25); however, this is also a very clear difference [49,50].

### 3.3. Texture Parameter Analysis

Hardness and crispness are textural properties that attract significant attention in the evaluation of baked goods because of their close association with human perceptions of freshness. It is desirable that these parameters are as low as possible [79]. There was an increase (*p* < 0.05) in the hardness in the case of more than 50% of chickpea flour share in the cookies (Table 4). Moreover, cookies without wheat flour (S100) were characterized as having more than 60% higher hardness (9.93 N) than wheat flour cookies—6.16 N (S0). Comparable results were observed by Chandra et al. [57], who reported increased hardness in biscuits with increasing levels of pearl millet flour up to 50%. The crispness did not differ significantly (*p* > 0.05), regardless of the flour used in the formulas; however, with increasing amounts of chickpea flour, the crispness increased slightly from 0.17 in the sample S0 up to 0.19 in the S100 sample. Similar results were reported by Lu et al. [39], who found increasing hardness of cookies with increasing share of chickpea flour, and Delgado-Andrade et al. [80], who found that a 30% share of chickpea flour of different varieties increased hardness of cookies to different levels, with a minimum of double. In turn, Schouten et al. [76] noticed a decrease in cookie hardness and an increase in crispness when the amount of chickpea flour was from 40% to 60%, while cookies containing 20% of chickpea flour did not differ from wheat cookies (*p* > 0.05).

### 3.4. Nutrient Composition

The increasing share of chickpea flour in shortbread cookies resulted in an increase (*p* < 0.05) in the protein content—by 50% in S100 in comparison to S0 (Table 5). Thongram et al. [28] found a significant (*p* < 0.05) increase in protein content (approx. 20% of protein content) in cookies after replacing 25% of wheat flour with chickpea flour. Chickpea flour contained almost twice as much protein as wheat flour, and almost four times as much fat (Table S2). Protein content in chickpeas range between 18.5 and 27 g/100 g, depending on the genotype, variety, and growth conditions, usually 20–24 g/100 g [81,82,83,84]. Chickpea flour contains 18.5–22.4 g/100 g, while wheat flour contains 10–12 g/100 g of proteins [81]. Flours differ not only in amount of protein, but also protein composition. While gliadin and glutenin (which form gluten with water) are the most important wheat proteins (together about 79% of proteins), in chickpea, prolamins are present only at the level of 3–7%, glutelins 18–24%, and the main proteins are globulins (53–60%), vicilin, and legumin [82]. Chickpea proteins show, among others, emulsifying properties, texture-forming properties, and high water holding capacity (WHC) [81,85]. Moreover, they are rich in lysine, unlike wheat. Lysin content in instant noodles enriched with 25% of chickpea flour was 44% higher than in wheat noodles [33].

Shortbread cookies are characterized by high content of fat—approx. 30%. S0 and S25 did not differ significantly, an but increase of about 20% was noticed in cookies containing 75% of chickpea flour (Table 5). Chickpea flour contained almost four times the fat of wheat flour (Table S2). Other researchers found an increase in fat level of 12% when 25% of wheat flour was replaced [28].

Total carbohydrate content in cookies containing up to 50% of chickpea flour did not differ significantly from wheat flour. Only a high share of chickpea flour caused about a 20% decrease in carbohydrate content. However, taking into account total fiber content, the digestible carbohydrate content (starch and sugars) decreased significantly with chickpea flour share (Table 5). Starch content in chickpeas depends on the variety and ranges between 49% and even 66% [82] in chickpea flour 40–42% [86] and 42–47 g/100 g d.m. according to Pournaki et al. [84] in comparison to about 72% in wheat flour [81].

The ash content increased significantly in S25, and it was even five times higher in S100 than in S0. This was a result of the difference in ash content in flours, which was more than six times higher in chickpea flour (0.45 g/100 g and 3.15 g/100 g, respectively). These findings are in agreement with results of other researchers. Fat content increased by 12%. Chickpea seeds, depending on the variety, contain 1.85–2.9 g of ash/100 g w.m. according to some authors [81,86], and even 2.69–3.90 g of ash/100 g d.m. [6,82]. In comparison, wheat flour for all uses, usually used in cookie production, contains 0.45% ash [81], as with the flour used in the present study.

All shortbread cookies were characterized by low moisture (11–12%) and did not differ (*p* > 0.05). Because of the low water activity, they are stable products when stored in low humidity.

Chickpea flour was characterized by about 10% higher water absorption capacity and 40% higher oil absorption capacity than wheat flour. However, the oil absorption capacity of flour used in our research was higher than that reported by DeAngelis et al. [11].

Similar results were obtained by Lu et al. [39], studying the effect of chickpea flour (10–40%) on biscuit quality. They found that protein and fat content increased, and starch content decreased significantly with increasing share of chickpea flour. Moreover, they showed a slowing of the digestion of starch, which lowers the glycemic index of biscuits. However, they found that 30% was the optimal level of chickpea content in terms of organoleptic characteristics.

### 3.5. Fiber Content

The content of soluble fiber (SDF) did not differ significantly (*p > 0.05*) in cookies containing wheat flour, regardless of the proportion of chickpea flour (Table 5). Only the S100 sample contained a slightly higher amount of SDF. In turn, the insoluble dietary fiber (IDF) content increased with the share of chickpea flour: even the use of 25% of chickpea flour instead of wheat flour resulted in a twofold increase in the amount of IDF, while 100% of the chickpea flour content resulted in a threefold increase. The total dietary fiber (TDF) content also gradually increased: in S25, TDF was 50% higher when in chickpea flour cookies S100 and almost 130% higher than in wheat cookies. According to Thongram et al. [28], replacement of 25% wheat flour with chickpea flour caused a 35% increase in total fiber content.

### 3.6. Bioactive Compound Content and Antioxidant Activities

The increase in the share of chickpea flour in shortbread cookies caused an increase in total polyphenol content (TPC), which was 2.5 times higher (534.7 µg GAE/g) in S100 than in S0 (207.9 µg GAE/g). Total flavonoid content (TFC) increased from 0.5 µg QE/g in S0 to 7.6 µg QE/g in S75, and up to 26.5 µg QE/g in S100 (Table 6). It was an effect of increasing share of chickpea flour because the total polyphenol content in chickpea flour was 60% higher than in wheat flour; however, total flavonoid content was not statistically different (*p* > 0.05) (Table S2). Thongram et al. [28] showed that replacing 25% of wheat flour by chickpea flour gave a polyphenol content twice as high, which turned out to be the highest (together with the sponge cake containing legume flour blend) among the tested flours from other legumes. Delgado-Andrade et al. [80] found a 30–50% higher content of total phenolic compounds in chickpea biscuits than in wheat biscuits.

Content of total carotenoid (TC) in wheat cookies was slightly lower than in wheat flour (4.3 and 5.1 g/100 g, respectively). The incorporation of chickpea flour significantly increased (*p* < 0.05) the TC content 2.4-fold (S25) up to 5-fold (S100) in the range from 10.17 μg/100 g up to 21.7 µg/100 g in chickpea cookies. It resulted from carotenoid content in chickpea flour, which was eight times higher than in wheat flour. A similar content of carotenoids in chickpea flour (46.3 μg/100 g) was found by Jukanti et al. [5].

The content of bioactive compounds resulted in an increase in the antioxidant activity of the cookies. Trolox equivalent antioxidant capacity (TEAC) increased fivefold, from 1.02 to 5.71 µmol TE/g, and DPPH radical scavenging activity (RSA) and ferric reducing antioxidant power (FRAP) increased sixfold, from 0.102 to 0.616 µmol TE/g and from 13.6 to 87.0 µmol TE/g, respectively. Even the lowest share of chickpea flour (25%) caused significant increases (*p* < 0.05) in all these parameters. The largest increase was recorded in the case of Fe^2+^ chelating ability (FCA), where the use of 25% of chickpea flour resulted in a threefold increase (0.036 to 0.119 µmol EDTA/g in S0 and S25, respectively), and with each increase of 25% of flour resulted in a two- to threefold increase in FCA to obtain finally 1.121 µmol EDTA/g in S100. Other authors found that free radical scavenging ability was about 70% higher in biscuits containing 25% of chickpea flour than in wheat cakes, and only cowpea flour caused higher RSA [24]. Delgado-Andrade et al. [80] found the antioxidant activity, determined as ABTS scavenging ability and FRAP, increased from 50% up to sixfold, depending on the chickpea flour origin (among two commercial and two non-commercial flours studied). Antioxidant activity of S100 cookies in the present study was higher than results reported by Delgado-Andrade et al. [80] for cookies from commercial flours and much lower than cookies made from non-commercial flours.

Generally, high antioxidant activity of chickpea cookies is strongly correlated with properties of flours used for the production. Radical scavenging activity of chickpea flour was about 2.5 times higher than wheat flour. This was in agreement with Thongram et al. [28], who reported that chickpea biscuits show double the antioxidant activity of chickpea flour, but in the case of wheat cookies, this was only 10%. However, they did not find significant differences in the DPPH radicals scavenging ability of wheat and chickpea flours. In turn, Segev et al. [87] stated that chickpea seeds of 17 cultivars (of Desi type and Kabuli type) differed in antioxidant activities.

Such a significant increase in antioxidant activity could result primarily from the higher content of polyphenols in chickpea flour, showing antioxidant activity, mainly isoflavones (biochanin A, formonentin, genistein and daidzein), phenolic acids (cinnamic acid, salicylic acid, hydroxycinnamic acid, *p*-coumaric acid, gallic acid, caffeic acid, vanillic acid, ferulic acid, anise acid, tannic acid, isoferulic acid, piperonyl, and chlorogenic acid), flavonols (quercetin, kaempferol, rutin and their derivatives) and other flavonoids (catechin, epicatechin and naringenin) [8,9,88,89]. This is also influenced by the higher content of protein with a high content of amino acids and peptides, which also play an important role in the formation of Maillard reaction products [6,9,76]. Moreover, it may be an effect of cookie exposure to high temperature due to the possibility of polyphenol damage and the formation of Maillard reaction products, the intensity of which depends on the temperature and heating time [90,91]. In the chickpea flour bread studied by Seveg et al. [91], TPC and FRAP of the crust increased in comparison to crumb and dough.

The results obtained confirm the results obtained by Thongram et al. [28], who used a 25% replacement of wheat flour with legume flours and showed that chickpea flour increase the polyphenol content in cookies almost twofold and the free radical scavenging capacity of DPPH from 22% to 42%. Application of the blend of 60% of wheat flour and four other legume flours (10% each) caused a much lower effect.

### 3.7. Hydroxymethylfurfural (HMF) Content

Hydroxymethylfurfural (HMF) is formed by dehydration of reducing sugars. One of the Maillard reaction products [92], it was the lowest in wheat cookies. The addition of chickpea flour up to 50% caused increased HMF content (from 17.5 µg/100 g in S0 to 95.6 in S50 µg/100 g); however in S75 and S100, HMF content was lower than in S50 (Table 6). Increased HMF content in S25 and S50 might have resulted from the higher content of sugars in chickpea flour, especially fructose [93]. This monosaccharide has a lower melting point than other sugars, so it is unstable in high temperatures [94]. The reduction in the HMF content in cookies in which the chickpea flour content was dominant (S75, S100) resulted from an increase in protein content. This led to a reduction in the reducing sugar/protein ratio in cookies, which is decisive in the formation of HMF in bakery products, especially short-crust cookies [95]. However, the HMF level in the cookies was not high compared to other studies, where HMF levels from 300 µg/100 g to 4000 µg/100 g were found in baked goods [92,96]. Ameur et al. [97] studied HMF content in 17 commercial cookies, differing in sugar content (from 5% to 77%) and fat content (from 8.7% to 29%), and found that HMF from 50 µg/100 g and even up to 7.46 mg/100 g. HMF formation is dependent on water activity, and it is significant in products of water activity lower than 0.4 [97,98]. Therefore, cookies from chickpea flour, which has higher water absorption capacity, have lower water activity and HMF concentration. Generally, HMF is believed to have carcinogenic effects at high concentrations; however, Moussou et al. [99] showed that HMF increases DPPH radical scavenging activity. Trace amounts of HMF were detected only in wheat flour (Table S2).

### 3.8. Phytates

Among the antinutritional ingredients contained in wheat flour and chickpea flour, thermolabile compounds probably do not play a negative role because they are inactivated by thermal treatment as baking. The content of phytates and tannins is reduced compared to grain due to dehulling and soaking and roasting used in the production of chickpea flour [11]. Both flours as well as the shortbread cookies showed the presence of phytates (Table S2 and Table 6), which reduce bioavailability of magnesium, zinc, and iron [11]. However, because phytates bind divalent metal ions in the digestive system and inhibit the action of amylases, they slow the digestion of carbohydrates and consequently reduce the glycemic index of chickpea products [9].

Flours did not differ in phytate content (*p* > 0.05), although according to Gadallah and Aljebreen [100], chickpea flour contains more phytic acid antinutritional compounds than wheat flour (type for all uses).

### 3.9. Polyphenol Profile

The chromatograms of cookies with the addition of chickpea flour showed more peaks and their area and height increased with the addition of flour (Figure S2). Changes in the identified compound content are presented in Table 7.

Polyphenol profiles of flours showed that chickpea flour contained much more protocatechuic acid, chlorogenic acid, 3-hydroxybenzoic acid, and epicatechin than wheat flour (Table S3). Only quercetin was present in lower amounts, and gallic acid was not detected in chickpea flour. In wheat flour, catechin, hippuric acid, 4-hydroxybenzoic acid, and caffeic acid were not detected. In contrast, Fratianni et al. [101] found gallic acid in two tested chickpea varieties from southern Italy, and Quintero-Soto et al. [102] found it in eighteen varieties.

In wheat cookies, there were no catechins, hippuric acid, or caffeic acid detected, which were found in cookies containing chickpea flour (Table 7). (+) Catechin levels increased fourfold when the share of chickpea flour increased from 25% to 50%, and up to about 15-fold in all-chickpea flour. In turn, quercetin was not detected in S75 or chickpea cookies, and in S50, it was lower than in S0 and S25.

### 3.10. Principal Component Analysis

**Figure 4:**
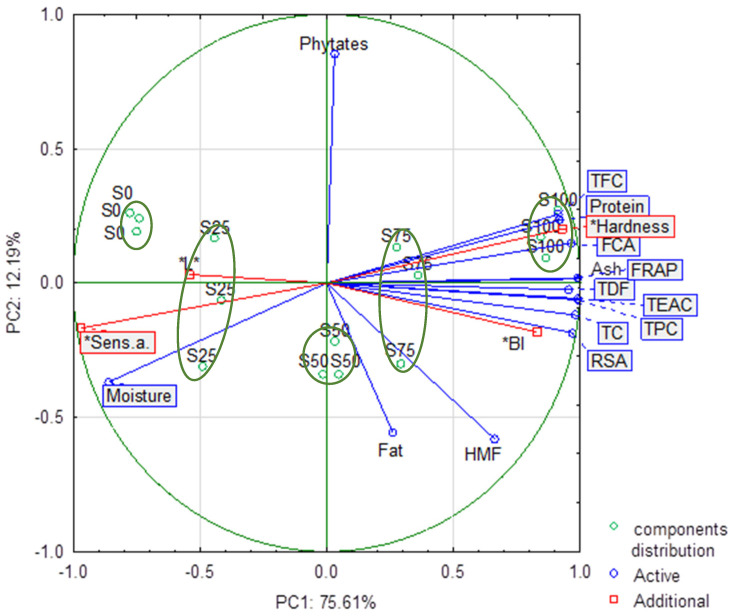
PCA biplot of the first two principal components for antioxidant activity, sensory assessment, hardness, color parameters, and shortbread cookie components and component distribution. TPC—total phenolic compound; TFC—total flavonoid compound; TC—total carotenoids; TEAC—Trolox equivalent antioxidant capacity; FRAP—ferric reducing antioxidant power; RSA—radical scavenging ability; FCA—ferrous chelating ability; Sens. a.—sensory assessment; L*—lightness, BI—browning index. * additional variables.

Principal component analysis (PCA) demonstrated that the initial two components explained 87.80% of the overall variance (Figure 4), while PC1 explained 75.61%. The strongest correlation in the first factor was found between TEAC, FRAP, and TPC, and ash content when the second factor did not show a strong correlation. No correlation was found between fat, phytate, and HMF content and other compounds.

The analysis of the corresponding score plot showed a clear separation of the samples along PC1 dependent on the different ratios between wheat and chickpea flour in the formulation. Compared to shortbread cookies made from wheat flour (S0), replacement of 25% with chickpea flour (S25) did not show distinct changes in physicochemical parameters. However, samples S0 and S100 were in different quartiles and differed significantly (*p <* 0.05). The protein content, total dietary fiber, ash content, TPC, TFC, TC, antioxidant activity (TEAC, RSA, FRAP, FCA), and hardness showed high contributions in the positive quadrant of PC1, being correlated with 75% and 100% chickpea flour cookies. Only phytates and fat content were not influenced by chickpea flour content. The component distribution confirmed that wheat cookies and chickpea cookies were placed in different quartiles and differed significantly, as other authors showed [28,80].

## 4. Conclusions

One way to increase chickpea consumption is to use chickpea flour instead of wheat flour in popular products. However, the biggest challenge is to replace wheat flour in bakery and confectionery products, in which gluten plays a significant technological role.

Chickpea flour seems to be a good replacement for wheat flour, but it is not suitable for all products because of different texture-forming abilities due to the lack of gluten.

The partial and complete replacement of wheat flour with chickpea flour in shortbread cookies gave products with good sensory properties and favorable physicochemical properties. The addition of 25% chickpea flour did not negatively affect cookie properties, but improved their antioxidant activity. Further increasing the share of chickpea flour had a positive effect on the protein content and antioxidant properties in shortbread cookies, where the level of proteins increased about 50% and the antioxidant properties were three to six times higher than in wheat cookies. Cookies containing up to 75% of chickpea flour were assessed as very good or good quality. Only cookies without wheat flour were assessed as sufficient quality. It could be concluded that chickpea flour can completely replace wheat flour in shortbread cookies, but the sensory properties would be worse. It could be concluded that part of the wheat flour content in shortbread cookies can be replaced by chickpea flour. Application of chickpea flour of 25% increases physicochemical properties without changes in sensory properties. There is up to 75% lower sensory quality, but increased antioxidant properties. However, complete replacement of wheat flour in shortbread cookies without changing the recipe resulted in slightly lower sensory quality. The availability of cookies containing chickpea flour to consumers may increase chickpea consumption. Further work should be carried out on the development of health-promoting products made from chickpea flour without added sugar and hydrogenated fats.

## Figures and Tables

**Table 1:** Ingredients used for shortbread cookie production (S—chickpea flour share from 0 to 100%).

	Control 0	Shortbread Cookies with Chickpea Flour			
		25%	50%	75%	100%
Wheat flour [g]	150	112.5	95	37.5	0
Chickpea flour [g]	0	37.5	95	112.5	150
Icing sugar [g]	50	50	50	50	50
Margarine [g]	100	100	100	100	100
Eggs [it] *	1	1	1	1	1
Salt [g]	0.25	0.25	0.25	0.25	0.25
Sample code	S0	S25	S50	S75	S100

* [it]—item.

**Table 2:** The effect of chickpea flour on sensory assessment of shortbread cookies (where 1.0—lowest note, 5.0—highest note for features; 1.0—lowest note, 9.0—highest note for overall desirability).

	Shortbread Cookies				
	S0	S25	S50	S75	S100
Appearance	4.8 ^a^ ± 0.45	4.8 ^a^ ± 0.45	4.4 ^ab^ ± 0.45	4.2 ^ab^ ± 0.45	3.6 ^c^ ± 0.55
Color	4.6 ^a^ ± 0.55	4.4 ^a^ ± 0.58	4.4 ^a^ ± 0.55	4.0 ^a^ ± 0.00	4.0 ^a^ ± 0.00
Taste	5.0 ^a^ ± 0.00	5.0 ^a^ ± 0.00	4.2 ^b^ ± 0.45	4.0 ^b^ ± 0.00	3.4 ^c^ ± 0.55
Odor	5.0 ^a^ ± 0.00	4.4 ^ab^ ± 0.55	4.2 ^b^ ± 0.45	4.0 ^b^ ± 0.00	3.6 ^c^ ± 0.55
Texture	4.6 ^a^ ± 0.55	4.4 ^a^ ± 0.55	4.4 ^a^ ± 0.55	4.0 ^a^± 0.00	3.6 ^b^ ± 0.55
Overall quality	4.82 ± 0.41 very good	4.62 ± 0.48 very good	4.30 ± 0.48 good	4.02 ± 0.20 good	3.24 ± 0.49 sufficient
Overall desirability	9.0 ^a^ ± 0.00	7.8 ^b^ ± 0.45	7.8 ^b^ ± 0.45	7.0 ^c^ ± 0.00	5.8 ^d^ ± 0.45

^a,b,c,d^ Means ± SD in rows with the same lowercase letter do not differ significantly (*p* < 0.05).

**Table 3:** The effect of chickpea flour on shortbread cookies (surface) color parameters (L*—lightness, a*—redness, b*—yellowness, C*—saturation, h*—tone angle, *ΔE*—overall color difference between samples S25—S100 and the control sample, BI—browning index).

	Shortbread Cookies				
	S0	S25	S50	S75	S100
L*	58.9 ^b^ ± 1.7	67.6 ^b^ ± 0.8	55.6 ^b^ ± 3.6	55.8 ^b^ ± 1.9	49.7 ^a^ ± 1.1
a*	11.1 ^a^ ± 0.5	10.6 ^a^ ± 0.5	15.4 ^b^ ± 1.3	16.9 ^b^ ± 0.6	16.6 ^b^ ± 0.4
b*	22.2 ^a^ ± 0.5	23.0 ^a^ ± 0.5	28.2 ^b^ ± 4.9	28.2 ^b^ ± 0.9	31.1 ^b^ ± 1.0
C*	24.8 ^a^ ± 0.6	24.2 ^a^ ± 0.6	32.3 ^b^ ± 4.9	32.9 ^b^ ± 1.1	35.3 ^b^ ± 0.8
h*	63.3 ^a^ ± 0.8	74.2 ^b^ ± 0.8	61.2 ^a^ ± 3.0	59.1 ^a^ ± 0.4	61.8 ^a^ ± 1.2
*ΔE*	-	8.01 ^a^ ± 0.60	11.10 ^b^ ± 1.00	14.42 ^c^ ± 0.21	12.25 ^d^ ± 0.59
BI	58.73 ^a^ ± 3.75	48.65 ^b^ ±1.17	98.52 ^c^ ± 6.54	105.12 ^c^ ± 1.63	100.70 ^c^ ± 1.95

^a,b,c,d^ Means ± SD in rows with the same lowercase letter do not differ significantly (*p* < 0.05).

**Table 4:** The effect of chickpea flour on texture analysis parameters of shortbread cookies.

	Shortbread Cookies				
	S0	S25	S50	S75	S100
Hardness [N]	6.16 ^a^ ± 0.06	6.92 ^ab^ ± 0.09	7.31 ^b^ ± 0.11	8.33 ^c^ ± 0.10	9.93 ^d^ ± 0.13
Crispness [N]	0.17 ^a^ ± 0.01	0.18 ^a^ ± 0.03	0.18 ^a^ ± 0.01	0.19 ^a^ ± 0.02	0.19 ^a^ ± 0.04

^a,b,c,d^ Means ± SD in rows with the same lowercase letter do not differ significantly (*p* < 0.05).

**Table 5:** The effect of chickpea flour on composition, fiber content, and water activity of shortbread cookies.

	Shortbread Cookies				
	S0	S25	S50	S75	S100
Moisture [g/100 g]	11.61 ^a^ ± 1.10	12.63 ^a^ ± 1.61	12.04 ^a^ ± 0.29	11.48 ^a^ ± 0.43	10.93 ^a^ ± 0.31
Protein [g/100 g]	7.35 ^a^ ± 0.14	7.30 ^ab^ ± 0.78	8.13 ^b^ ± 0.30	10.35 ^c^ ± 0.07	11.16 ^d^ ± 0.67
Fat [g/100 g]	28.99 ^b^ ± 0.31	29.02 ^b^ ± 0.12	28.53 ^a^ ± 0.14	35.26 ^c^ ± 0.04	37.04 ^d^ ± 0.02
Carbohydrates [g/100 g]	51.74 ^c^ ± 1.11	50.5 ^c^ ± 0.4	50.39 ^c^ ± 0.51	41.68 ^b^ ± 0.21	39.27 ^a^ ± 0.42
Starch and sugars [g/100 g]	46.67 ^e^ ± 0.57	43.04 ^d^ ± 0.24	37.96 ^c^ ± 0.21	32.79 ^b^ ± 1.15	27.79 ^a^ ± 0.26
Ash [g/100 g]	0.31 ^a^ ± 0.01	0.56 ^b^ ± 0.09	0.91 ^c^ ± 0.06	1.23 ^d^ ± 0.12	1.60 ^e^ ± 0.05
IDF [g/100 g]	2.48 ^a^ ± 0.25	5.07 ^b^ ± 0.40	5.85 ^c^ ± 0.81	6.29 ^d^ ± 1.53	8.65 ^e^ ± 0.74
SDF [g/100 g]	2.55 ^a^ ± 0.28	2.50 ^a^ ± 0.24	2.69 ^a^ ± 0.10	2.62 ^a^ ± 0.18	2.86 ^b^ ± 0.08
TDF [g/100 g]	5.03 ^a^ ± 0.53	7.56 ^b^ ± 0.16	8.54 ^c^ ± 0.71	8.91 ^c^ ± 1.35	11.51 ^d^ ± 0.66
Water activity	0.234 ^c^ ± 0.001	0.244 ^d^ ± 0.001	0.257 ^e^ ± 0.001	0.204 ^b^ ± 0.003	0.168 ^a^ ± 0.001

IDF—insoluble dietary fiber; SDF—soluble dietary fiber; TDF—total dietary fiber. ^a,b,c,d,e^ Means ± SD in rows with the same lowercase letter do not differ significantly (*p* < 0.05).

**Table 6:** The effect of chickpea flour on phenolic compounds, antioxidant properties and phytates of shortbread cookies.

	Shortbread Cookies				
	S0	S25	S50	S75	S100
TPC [µg GAE/g]	207.9 ^a^ ± 4.6	273.6 ^b^ ± 3.7	383.7 ^c^ ± 13.7	453.6 ^d^ ± 2.2	534.7 ^e^ ± 2.7
TFC [µg QE/g]	0.51 ^a^ ± 0.16	1.79 ^b^ ± 0.16	5.27 ^c^ ± 0.27	7.56 ^d^ ± 0.32	26.52 ^e^ ± 0.63
TCC [µg/100 g]	4.30 ^a^ ± 0.100	10.17 ^b^ ± 0.153	14.17 ^c^ ± 0.115	18.37 ^d^ ± 0.153	21.67 ^e^ ± 0.250
TEAC [µmol TE/g]	1.02 ^a^ ± 0.14	1.95 ^b^ ± 0.16	3.64 ^c^ ± 0.29	4.44 ^d^ ± 0.09	5.71 ^e^ ± 0.24
FRAP [µmol TE/g]	13.6 ^a^ ± 0.6	29.4 ^b^ ± 0.4	44.7 ^c^ ± 0.7	66.6 ^d^ ± 0.6	87.0 ^e^ ± 1.2
RSA [µmol TE/g]	0.102 ^a^ ± 0.004	0.279 ^b^ ± 0.009	0.410 ^c^ ± 0.003	0.560 ^d^ ± 0.056	0.616 ^e^ ± 0.028
FCA [µmol EDTA/g]	0.036 ^a^ ± 0.008	0.119 ^b^ ± 0.027	0.349 ^c^ ± 0.013	0.522 ^d^ ± 0.030	1.121 ^e^ ± 0.041
Phytates [mg PA/g]	0.030 ^a^ ± 0.005	0.035 ^a^ ± 0.005	0.036 ^a^ ± 0.009	0.035 ^a^ ± 0.004	0.037 ^a^ ± 0.005
HMF [µmol/100 g]	17.46 ^a^ ± 1.05	32.81 ^b^ ± 3.29	95.65 ^c^ ± 2.57	75.51 ^d^ ± 1.77	64.43 ^e^ ± 2.65

TPC—total phenolic compound; GAE—gallic acid equivalent; TFC—total flavonoid compound; TCC—total carotenoid content; QE—quercetin equivalent; TEAC—Trolox equivalent antioxidant capacity; TE—Trolox equivalent; FRAP—ferric reducing antioxidant power; RSA—radical scavenging ability; FCA—ferrous chelating ability; EDTA—ethylenediaminetetraacetic acid; PA—phytic acid; HMF—hydroxymethylfurfural. ^a,b,c,d,e^ Means ± SD in rows with the same lowercase letter do not differ significantly (*p* < 0.05).

**Table 7:** Phenolic compound profile in shortbread cookies containing chickpea flour [mg/100 g].

	S0	S25	S50	S75	S100
gallic acid mg/100 g	4.14 ^a^ ± 0.30	4.08 ^a^ ± 0.13	7.64 ^b^ ± 0.29	9.41 ^c^ ± 0.10	13.33 ^d^ ± 0.38
protocatechuic acid mg/100 g	3.04 ^a^ ± 0.24	4.17 ^b^ ± 0.29	7.62 ^c^ ± 0.15	10.95 ^d^ ± 0.77	18.46 ^e^ ± 0.29
chlorogenic acid mg/100 g	48.00 ^a^ ± 1.58	63.58 ^b^ ± 1.41	114.5 ^c^ ± 4.1	206.8 ^d^ ± 12.1	2378.7 ^e^ ± 19.1
(+) catechin mg/100 g	nd	34.00 ^a^ ± 1.88	144.1 ^b^ ± 2.1	291.9 ^c^ ± 7.5	540.7 ^d^ ± 21.3
hippuric acid mg/100 g	nd	2.54 ^a^ ± 0.10	6.61 ^b^ ± 0.28	11.49 ^c^ ± 0.26	26.42 ^d^ ± 0.35
caffeic acid mg/100 g	nd	nd	0.135 ^a^ ± 0.002	0.228 ^b^ ± 0.025	0.309 ^c^ ± 0.031
(−) epicatechin mg/100 g	1.43 ^b^ ± 0.27	1.26 ^b^ ± 0.07	0.621 ^a^± 0.087	1.08 ^b^ ± 0.12	2.46 ^c^ ± 0.13
3-hydroxybenzoic acid mg/100 g	3.24 ^b^ ± 0.34	2.72 ^a^ ± 0.07	5.49 ^c^ ± 0.05	7.48 ^d^ ± 0.31	15.48 ^e^ ± 0.18
*p*-coumaric acid mg/100 g	0.145 ^c^ ± 0.034	0.092 ^a^ ± 0.004	0.077 ^a^ ± 0.008	0.080 ^a^ ± 0.009	0.118 ^b^ ± 0.013
quercetin mg/100 g	0.124 ^b^ ± 0.003	0.124 ^b^ ± 0.007	0.087 ^a^ ± 0.007	nd	nd

nd—not detected, ^a,b,c,d,e^ means ± SD in rows with the same lowercase letter do not differ significantly (*p* < 0.05).

## Data Availability

The original contributions presented in the study are included in the article/Supplementary Material, further inquiries can be directed to the corresponding author.

## Supplementary Materials

The following supporting information can be downloaded at https://www.mdpi.com/article/10.3390/foods13152356/s1. Figure S1: Wheat flour (A) and chickpea flour (B) used for shortbread cookie preparation; Table S1: Objective color parameters of flours used in the production of shortbread cookies (*L**—lightness, *a**—redness, *b**—yellowness, *C**—saturation, *h**—hue); Table S2: Approximate composition and properties of wheat flour and chickpea flour used in the study; Table S3: Content of phenolic compounds in wheat and chickpea flours (mg/100 g); Figure S2: Chromatogram of chickpea flour content in cookies on their phenolic compound profiles (black line—S0, blue—S25, pink—S50, red—S75, green—S100), λ = 280 nm; 1—gallic acid, 2—protocatechuic acid, 3—(+) catechin, 4—chlorogenic acid, hippuric acid, (−) epicatechin, 7—3-hydroxybenzoic acid.
