# Supplementary material for: Effect of Chickpea (Cicer arietinum L.) Flour Incorporation on Quality, Antioxidant Properties, and Bioactive Compounds of Shortbread Cookies
Source: Foods. 2024 Jul 26;13(15):2356. doi: 10.3390/foods13152356 (PMC11311373; doi:10.3390/foods13152356)
Supplement: Supplementary file 1 [file foods-13-02356-s001.zip › Supplementary materials FV.pdf]

Supplement to Article

Suplementary materials to

Effect of Chickpea (*Cicer arietinum* L.) Flour Incorporation on Quality, Antioxidant Properties, and Bioactive Compounds of Shortbread Cookies

Katarzyna Felisiak [1,*], Sylwia Przybylska [1], Grzegorz Tokarczyk [1], Małgorzata Tabaszewska [2], Jacek Słupski [2], and Joanna Wydurska [1]

A) B)

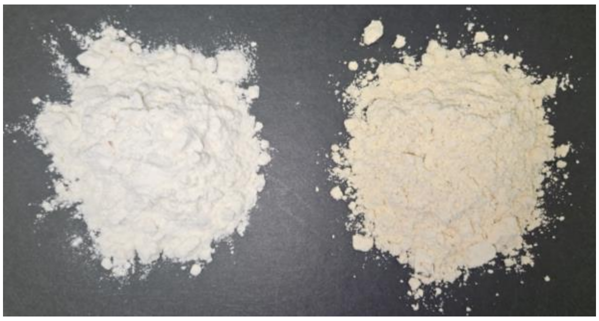

Figure S1. Wheat flour (A) and chickpea flour (B) used for shortbread cookies preparation.

Table S1. Objective color parameters of flours used in the production of shortbread cookies (L^* - lightness, a^* - redness, b^* - yellowness, C^* - saturation, h^* - hue).

	**Color parameters**				
**Flours**	L^*	a^*	b^*	C^*	h^*
Wheat	$88.9^a \pm 0.50$	$1.5^a \pm 0.10$	$10.1^a \pm 0.08$	$10.2^a \pm 0.09$	$81.3^a \pm 0.52$
Chickpea	$85.8^b \pm 0.16$	$3.5^b \pm 0.06$	$19.2^b \pm 0.06$	$19.5^b \pm 0.06$	$79.7^b \pm 0.17$

[a,b]Means ± SD in columns with the same lower case letter do not differ significantly ($P < 0.05$)

Table S2. Proximate composition and properties of wheat flour and chickpea flour used in the study.

	**Wheat flour**	**Chickpea flour**
Moisture [g/100 g]	9.45[a] ± 0.93	8.61[a] ± 0.41
Protein [g/100 g]	10.88[a] ± 0.07	20.10[b] ± 0.23
Fat [g/100 g]	1.36[a] ± 0.09	5.95[b] ± 0.12
Carbohydrates [g/100 g]	77.86[a] ± 1.12	62.19[b] ± 0.99
Starch and sugars [g/100 g]	73.48[a] ± 0.95	44.16[b] ± 0.18
Ash [g/100 g]	0.451[a] ± 0.026	3.153[b] ± 0.031
IDF	1.97[a] ± 0.08	13.68[b] ± 0.90
SDF	2.41[a] ± 0.10	4.35[b] ± 0.45
TDF	4.38[a] ± 0.17	18.03[b] ± 0.81
TPC [mg GAE/g]	0.310[a] ± 0.009	0.520[b] ± 0.006
TFC [µg QE/g]	3.90[a] ±0.27	4.08[a] ±0.32
TC [µg/100g]	5.07[a] ±0.115	41.57[b] ±0.153
TEAC [µmol TE/g]	3.63[a] ± 0.05	4.89[b] ± 0.19
FRAP [µmol TE/g]	30.07[a] ± 1.79	32.69[a] ± 0.41
RSA [µmol TE/g]	0.169[a] ± 0.016	0.456[b]±0.021
Phytates [mg PA/g]	0.126[a] ± 0.011	0.132[a] ± 0.009
HMF [µg/100 g]	2.483 ± 0.101	nd
Water activity	0.567[a] ± 0.020	0.608[a] ± 0.022
Water absorption capacity [g/g]	0.666[a] ± 0.006	0.737[b] ± 0.001
Oil absorption capacity [g/g]	0.620[a] ± 0.099	0.880[b] ± 0.006

TPC - total phenolic compounds; GAE - gallic acid equivalent; TFC – total flavonoids compounds; TC – total carotenoids content; QE – quercetin equivalent; TEAC - Trolox equivalent antioxidant capacity; TE - Trolox equivalent; FRAP - Ferric reducing antioxidant power; RSA – Radical scavenging ability; FCA - ferrous chelating ability; EDTA - ethylenediaminetetraacetic acid; PA – phytic acid; HMF – hydroxymethylfurfural; IDF – insoluble dietary fiber; SDF – soluble dietary fiber; TDF – total dietary fiber;

[a,b] Means ± SD in row with the same lower case letter do not differ significantly ($P < 0.05$)

Table S3. Content of phenolic compounds in wheat and chickpea flours [mg/100 g]

	Wheat Flour	Chickpea Flour
Gallic acid mg/100 g	0.625 ± 0.008	nd
Protocatechuic acid mg/100 g	1.27[a] ± 0.01	3.68[b] ± 0.31
chlorogenic acid mg/100g	74.66[a] ± 1.28	490.0[b] ±10.8
(+) catechin mg/100 g	nd	374.8 ± 5.6
hippuric acid mg/100 g	nd	18.26 ± 0.24
4-hydroxybenzoic acid mg/100 g	nd	0.116 ± 0.011
caffeic acid mg/100 g	nd	0.075 ± 0.001
(-)epicatechin mg/100g	0.129[a] ± 0.014	2.85[b] ± 0.07
3-hydroxybenzoic acid mg/100g	0.751[a] ± 0.026	13.83[b] ±0.20
p-coumaric acid mg/100g	0.269[a] ± 0.022	0.107[b] ± 0.005
quercetin mg/100 g	1.91[a] ± 0.44	0.936[b] ± 0.058

nd – not detected

[a,b] Means ± SD in row with the same lower case letter do not differ significantly ($P < 0.05$)

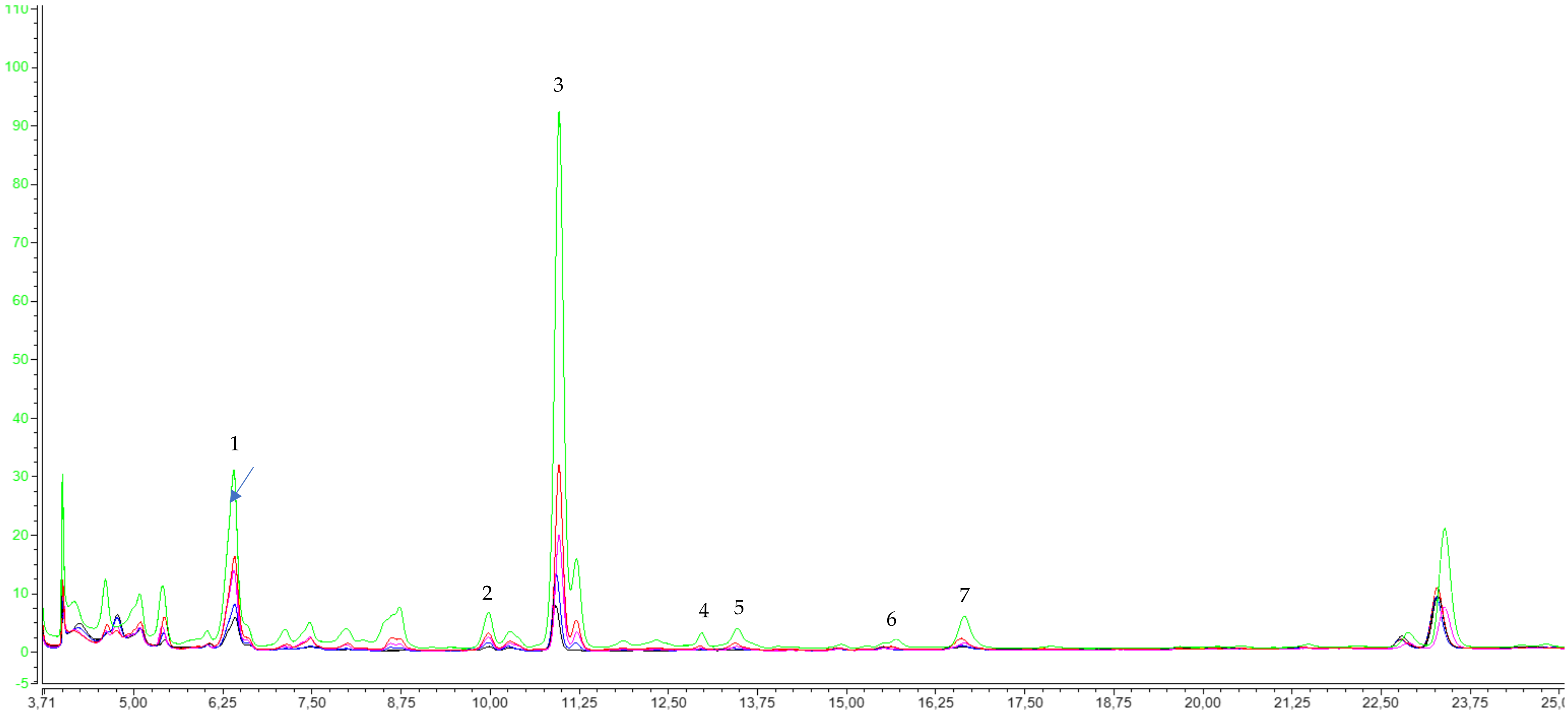

Figure S2. Chromatogram of chickpea flour content in cookies on their phenolic compounds profiles (black line – S0, blue – S25, pink – S50, red – S75, green - S100), λ = 280 nm; 1 - gallic acid, 2 - protocatechuic acid, 3 - (+) catechin, 4 - chlorogenic acid, hippuric acid, (-) epicatechin, 7 - 3-hydroxybenzoic acid.
